# Integrated primary care and social services for older adults with multimorbidity in England: a scoping review

**DOI:** 10.1186/s12877-021-02618-8

**Published:** 2021-12-03

**Authors:** Hajira Dambha-Miller, Glenn Simpson, Lucy Hobson, Paul Roderick, Paul Little, Hazel Everitt, Miriam Santer

**Affiliations:** 1 Primary Care Research Centre, University of Southampton, Aldermoor Health Centre, Aldermoor Close, Southampton, SO16 5ST UK; 2 Department of Population Health, University of Southampton, Southampton, UK

**Keywords:** Integrated care, Older adults, Multimorbidity, England

## Abstract

**Background:**

As the prevalence of older adults with multimorbidity increases, greater integration of services is necessary to manage the physical and psycho-social needs of this cohort. This study describes and summarises current evidence, clinical provision and progress towards integrated primary care and social services for older adults with multimorbidity in England.

**Methods:**

A scoping review was conducted involving systematic searches of a range of electronic academic and policy databases. Articles were screened and extracted in duplicate by two independent reviewers. Data were extracted onto a charting sheet and thematic synthesis was used to summarise findings. Articles were included if published in English and related to primary care, social care and multimorbidity in older adults in England. Conceptually, the review was framed using the Rainbow Model of Integrated Care.

**Results:**

The search yielded 7656 articles of which 84 were included. Three themes were identified: (1) a focus on individual level services rather than multi-level or multi-sector integration, with an increasing emphasis on the need to consider broader determinants of population health as critical to integrated care for older adults with multimorbidity; (2) the need for policymakers to allow time for integration to embed, to enable new structures and relationships to develop and mature; and (3) the inherent tension between top-down and bottom-up driven approaches to integrated care requires a whole-systems structure, while allowing for local flexibilities.

**Conclusions:**

There is limited evidence of multi-level and multi-sector integration of services for older adults with multimorbidity in England. The literature increasingly acknowledges wider determinants of population health that are likely to require integration beyond primary care and social services. Improving clinical care in one or two sectors may not be as effective as simultaneously improving the organisation or design across services as one single system of provision. This may take time to establish and will require local input.

**Supplementary Information:**

The online version contains supplementary material available at 10.1186/s12877-021-02618-8.

## Introduction

Closer health and social care integration has been a key policy goal of successive UK governments for 40 years but the advancement of this agenda has not been achieved at the pace required to meet the demands of an increasingly ageing population with higher levels of multimorbidity [1, 2]. Recent prediction forecasting of care dependency profiles suggests that 80% of the ageing population, that is people aged 65 years or older according to the UK National Health Service, will require medium or high dependency care due to multimorbidity [3]. In this context, it is essential that primary care is capable of working closely with social services and wider community care providers to harness collective capacity, which can address the range of behavioural, social, and physical health care needs of the population. This requires more careful consideration of the organisation, structures, systems and funding across providers to identify specific opportunities for successful integration. The scoping review adopted Leutz’s definition of integration ‘as the search to connect the healthcare system (acute, primary medical and skilled) with other human service systems (e.g. long-term care, education and vocational and housing services) to improve outcomes (clinical, satisfaction and efficiency)’. [4].

Given the substantial funding provided for integration pilots including a variety of testbeds, and the extensive research and evaluation in practice that has already been conducted [5], it is valuable to learn from these to understand current progress and anticipate future challenges to successful implementation. Whilst previous reviews have been conducted, these have mainly been limited to searches of the published literature, which may not adequately capture efforts in private or voluntary sector care organisations, where most social care is provided [6–9]. Moreover, few earlier studies have specifically examined the literature related to England in order to consider the contextual factors of this setting. There is also a relative paucity of evidence that considers integration in relation to primary care, where most care of older adults with multimorbidity occurs [7, 10]. To address these gaps in the evidence base, a scoping review was conducted to describe and summarise current evidence, clinical provision and progress towards integrated primary care and social services for older adults with multimorbidity in England.

## Methods

### Review approach and conceptual framework

The study followed the Preferred Reporting Items for Systematic Reviews and Meta-Analyses (PRISMA) guidelines for scoping reviews [11]. The scoping review allowed rapid mapping out of key existing work and current progress in this field. Conceptually, the review was framed using the Rainbow Model of Integrated Care, a framework that permits better definition and understanding of integrated care from a primary care perspective [12]. This multi-level conceptual framework describes dimensions that play inter-connected roles on the macro- (system integration), meso- (organisational and professional integration) and micro-level (clinical, service and personal integration), alongside dimensions (functional and normative integration) that enable the integration between different levels within a health system [13]. The model promotes the provision of continuous, comprehensive and coordinated care to the individual and population [12].

### Search strategy

A systematic electronic search was conducted in Medline, EMBASE, The Cochrane Library, Web of Science, the Cumulative Index to Nursing and Allied Health Literature, and Science and Social Science Citation Indices from database inception until the 16th June 2020. For searches of electronic databases, text and MeSH terms were limited to primary care, social services and older adults. Detailed search terms are available in supplementary file. Unpublished literature was searched for in Opengrey and through the websites of Clinical Commissioning Groups, GP federations, the Department of Health and Social Care, third sector bodies and private organisations who deliver social care. Hand searching of the bibliographies of included works and relevant systematic reviews for any additional relevant data was conducted. The views of topic experts and service users were sought to source further data. All articles identified were imported into Rayyan software for screening, which was conducted in blinding mode.

### Inclusion/exclusion criteria

Articles were eligible for inclusion if published in the English language and their content was primarily related to the key inclusion/exclusion criteria of primary care, social care and multimorbidity in older adults, specifically in England. Due to the broad aim of this scoping review, researchers adopted flexibility in study designs including newsletters, discussion papers, government reports, company reports, blogs, working papers, policy recommendations, webinars and dissertations. Quality assessment is not a priority for scoping reviews, therefore articles were not excluded on this basis.

### Study selection and data extraction

Titles and abstracts were screened with each article assessed for relevance according to the inclusion criteria. Full-text articles were retrieved. Both screening and data extraction were carried out independently by two reviewers. A data charting form was used, which was specifically designed according to the study conceptual framework described above. The level of integration within each included article was examined in line with the Rainbow Model of Integrated Care conceptual framework (i.e. macro, meso or micro-level and vertical/horizontal integration), article characteristics and key findings.

A list of data extracted on the charting form is summarised in Table 1 below. Any disagreement between reviewers about data was resolved through discussion until a consensus was reached.

**Table 1:** Summary of data charting form

• Title • Author • Year • Study design (RCT, Scoping Review or Case Study etc) • Geographical Location (Manchester, Norfolk or London, etc) • Setting (Social care, primary care or care home etc) • Sample type (GP, patient, social worker or relative, etc) • Source of data (Primary research, secondary analysis or commentary, etc) • Primary aim of study, research or document (As set out in study abstract) • Method (Questionnaire, semi-structured interviews or scoping review, etc) • Key findings/themes (As set out in study abstract) • Examples of integrated care provided (Any examples of integrated care practice, initiatives, structures, models, etc., e.g. Case Management Model, Chronic Care Model; Social Prescribing) • Clinical or practice integration (micro-level), e.g. the extent to which staff, management and patient records etc., are integrated • Organisational integration (meso-level), e.g. the extent to which integration of services has been achieved across different organisations • System integration (macro-level), e.g. the degree of alignment of rules and integration of policies within a health and social system.	

### Summarising and analysis

Researchers used counts to summarise article characteristics, and the charting technique to iteratively synthesise and interpret findings by sifting and sorting material [14, 15]. Repeated reference to the study conceptual framework was made during this process. Using thematic synthesis, key excerpts of extracted text were first coded by three members of the team (HDM, GS and SH). Deductive analysis was used in deriving a conceptual framework from the research aims and theory, while also seeking to inductively identify codes and themes from the synthesis of included articles. Initial codes were refined into themes. Members of the team experienced in systematic reviewing who had not previously been engaged in the coding process, were involved in the final stages of the analysis. This provided an additional perspective on the analytical process to strengthen both the quality and validity of the findings.

## Results

In total 7656 articles were identified including 6426 from electronic databases, 1118 via Opengrey and 112 from websites and experts. Following title and abstract screening, 809 articles underwent full-text screening, which resulted in a final 84 articles being included in the review. A flowchart of the screening process, including the reasons for exclusion, is shown in Supplementary material [16].

### Characteristics of included studies

The 84 included articles represented multiple locations across England, including regions in the south-west, north-east, north-west and Greater London. These were from a range of sectors; primary care, secondary care, social care, voluntary sectors, local government, local authority and public health. The most frequent study designs or types were qualitative (*n* = 18), followed by mixed-methods (*n* = 12), analysis/commentaries (*n* = 12), systematic/scoping/evidence reviews (*n* = 10), randomised controlled trials (*n* = 9), policy documents (*n* = 8), quantitative studies (*n* = 7), thesis (*n* = 4), editorials (*n* = 2) and books/book reviews (*n* = 2). Included articles were published between 1996 and 2020. The characteristics of included articles have been summarised in Table 2.

**Table 2:** Characteristics of included studies

Source	Setting	Aim	Results	Rainbow Model’s three levels (***micro, meso and macro***): Evidence of: 1, 2, 3 or none of the levels
Joint approaches for a better old age: developing services through joint commissioning. Poxton R. 1996.	Primary care. Secondary care. Local government.	Explores the strengths, weaknesses, barriers to joint commissioning of services from health and local authorities.	Difficult to define success/outcomes; significant change often takes longer than time of observation/assessment; high turnover of staff in social services.	3
Randomised controlled trial of follow up care in general practice of patients with myocardial infarction and angina: final results of the Southampton heart integrated care project (SHIP). Jolly K, Bradley F, Sharp S, Smith H, Thompson S, Kinmonth AL, Mant D. 1999.	Primary care. Secondary care.	Assess the effectiveness of a programme to coordinate and support follow-up care in general practice after a hospital diagnosis of myocardial infarction or angina.	Simply coordinating and supporting existing NHS care seems insufficient.	1
Exploratory cluster randomised controlled trial of shared care development for long-term mental illness. Byng R, Jones R, Leese M, Hamilton B, McCrone P, Craig T. 2004.	Primary care.	Determine the effects of Mental Health Link, a facilitation-based quality improvement programme to improve communication between the teams and systems of care within general practice.	Facilitated intervention tailored to context has the potential to improve care and interface working.	2
Contextualising the coordination of care in NHS trusts: An organisational perspective. Maxwell S. 2007.	Primary care.	Understand why change oriented towards improving the coordination of care for long term users of healthcare (and related) services is difficult to achieve. Identify possibilities for how these difficulties may be overcome.	Local and wider contextual factors in the NHS can fragment thinking about how care should be organised. For example, the character of the local electorate, the style and expected longevity of senior leadership, can undermine success in achieving coordinating care.	2
Integrating assessments of older people: examining evidence and impact from a randomised controlled trial. Clarkson P, Brand C, Hughes J, Challis D. 2011.	Care home sector.	Enable potentially treatable health conditions to be identified, which might obviate the need for care home admission given the proper intervention.	Integrating health and social care assessments offers benefits, principally a reduction in physical deterioration for the very frail.	1
Integration and the NHS reforms. Wistow G. 2011.	Local government. Primary care. Secondary care.	Analysing the implications of the Health and Social Care Bill and subsequent Act (2012) from a local government perspective.	Integration will be determined by whether the NHS is enabled to become a fuller part of the local family of public services and how far it remains a single-purpose, non-elected and nationally controlled service.	3
Redesigning the general practice consultation to improve care for patients with multimorbidity. Kadam U. 2012.	Primary care.	Discussion of changes to the standard primary care consultation for patients with multiple conditions.	Guided care model – Nurse-led coordination of interaction between the patient, primary care doctors, and healthcare teams. A trial found limited effectiveness in reducing healthcare use.	None.
Managing patients with mental and physical multimorbidity. Mercer FW, Gunn J, Bower P, Wyke S, Guthrie B. 2012.	Primary care.	Consider changes needed in managing the physical and mental health need of people with multimorbidity.	Application of the principles of chronic disease management to depression provides a potentially useful model for delivery of integrated care.	None.
Does Comprehensive Geriatric Assessment (CGA) have a role in UK care homes? Gordon, AL. 2012.	Care home sector.	Assess whether comprehensive geriatric assessment has a role in UK care homes.	Discontinuity of care between care home and GP; lack of anticipation; communication failure between GP and care home; inadequate training in care of older patients; arbitrary boundaries between care homes and NHS, which interfere with care.	2
National Evaluation of the Department of Health’s Integrated Care Pilots RAND Europe, Ernst & Young LLP. 2012.	Primary care.	Evaluation of the Integrated Care Pilot programmes.	Most pilots concentrated on horizontal integration, e.g. integration between community-based services such as general practices, community nursing services and social services, rather than vertical integration, e.g. between primary care and secondary care.	3
Still a fine mess? Local government and the NHS 1962 to 2012. Wistow G. 2012.	Primary care. Secondary care. Social services. Local Government.	Taking ‘a long view’ of initiatives promoting integration between local government and the NHS, with the objective of seeking to understand why they have achieved consistently disappointing results.	Barriers lie in the foundational principles of basing: (a) responsibilities on the skills of providers rather than service user need (b) organisational forms are based on separation rather than interdependence with national uniformity driving the NHS and local diversity driving local authorities.	2
Assistive technologies in caring for the oldest old: a review of current practice and future directions. Robinson L, Gibson G, Kingston A, Newton L, Pritchard G, Finch T, Brittain K. 2013.	Secondary care.	How can assistive technology support the future care of the oldest old.	Assistive technology has potential to help older people to ‘age in place’, but the ways in which assistive technologies are marketed to this group needs to be urgently addressed.	None.
Delivering better services for people with long-term conditions: Building the house of care. Coulter A, Roberts S, Dixon A. 2013.	Primary care. Social care.	Improve personalised services for individuals with long-term conditions.	Leadership from professional bodies is needed to drive a culture change of personalised care planning integrated across services.	2
Co-ordinated care for people with complex chronic conditions: Key lessons and markers for success. Goodwin N, Sonola L, Thiel V, Kodner D. 2013.	Primary care. Secondary care.	Identify strengths and weaknesses in five vanguard care co-ordination programmes.	Programmes of care co-ordination take years to grow into mature models of care. Therefore, success in care co-ordination is a long-term process, facilitated by key local leaders, during which the capability and legitimacy of new ways of working is built up over time.	3
New conversations between old players? The relationship between general practice and social care in an era of clinical commissioning. Glasby J, Miller R, Posaner R. 2013.	Primary care. Adult social care.	Examine evidence about joint work between general practice and adult social care in relation to older people.	More opportunities needed for GPs and social workers to understand their respective roles. A more rigorous approach to the setting and monitoring of outcomes will enable ideas to be tested out in practice. Sufficient time must be given to enable new structures to settle in and for trusting relationships to be developed.	2
Networks that work: Partnerships for integrated care and services. Langford K, Baeck P, Hampson M. 2013.	Primary care. Social care. Secondary care. Voluntary sector.	Show how third sector organisations can develop and provide services to encourage self-management.	Improving the capacity of patients to self-manage needs the development of clear services to provide extra capacity. Many people can be helped to do this on their own.	1
Evidence review - integrated health and social care. Institute of Public Care, Oxford Brookes University. 2013.	Care home sector. Secondary care. Reablement services. Primary care.	Understand the characteristics of effective workforce practice in integrated care services.	Much of the work identified was not primarily concerned with workforce issues and connections between workforce approaches, and the outcomes for service users were not always addressed. Creating new roles working across professional boundaries supports integrated delivery.	1
Rethinking the integration agenda. Stirling S, Malcolm A, Corbett-Nolan A. 2013.	Focused on integrated care generically.	Understand what the obstacles to integration are and offers practical ideas on next steps.	Reasons why care services have not developed further and faster include: Organisational Obstacles, Systemic Obstacles, Public Engagement and Politics and Policy.	2
A Question of Behaviours: Why delivering care integration and managing acute demand depends as much on changing behaviour as new systems and structures. Khaldi A. 2013.	Primary care. Secondary care. Community services.	This report addresses two connected challenges: the increasing dependency on acute settings and urgent care, particularly for the elderly ; the positive agenda to integrate care in home and community settings.	Structural ‘big system’ change alone will not work. Behavioural norms for professionals and the public are stronger than any new system can create.	3
Health and Care Integration Making the case from a public health perspective. Public Health England. 2013.	Public Health.	To help local areas, in particular health and wellbeing boards, make the case for integration focused on individuals’ health and wellbeing as well as their quality of life if they become sick.	The launch of the 14 integration pioneer sites, combined with the introduction of the Better Care Fund, creates the prospect for local areas to do things differently. The pioneers will implement innovative approaches and share experiences.	2
Making our health and care systems fit for an ageing population. Patterson L. 2014.	Multiple contexts.	To provide a high-level resource and reference guide for local service leaders who want to improve care for older people.	Transforming services for older people requires a fundamental shift towards care that is co-ordinated around the full range of an individual’s needs and care that prioritises prevention and support for maintaining independent living.	2
Delivering Integrated Care and Support. Petch A. 2014.	Primary care. Secondary care. Community care.	To review of research evidence on the factors that underpin best health and social care integrated practice.	There is a need to focus on the six key dimensions for effective implementation of integrated care and support: vision; leadership; culture; local context; integrated teams; time.	2
Integrated health and social care in England: the story so far. Royal College of Nursing. 2014.	Multiple contexts.	Overview of current integrated health and social care, the rationale behind care integration, facilitators and barriers to integrated working, and related workforce and funding issues.	Integrated care might be seen politically as a quick fix for the problems facing a health and social care system. Achieving real long-lasting change will take time, as well as prolonged effort to establish and maintain successful integrated working practices.	2
Enablers and barriers in implementing integrated care. Maruthappu M, Hasan A, Zeltner T. 2015.	No specific context.	Examine the fragmentation in patient services observed in many health care systems. This article discusses how integration may be achieved.	Implementing integrated care holds the potential for improving health care outcomes, access and significant financial savings.	3
Common patterns of morbidity and multi-morbidity and their impact on health-related quality of life: evidence from a national survey. Mujica-Mota RE, Roberts M, Abel G, Elliott M, Lyratzopoulos G, Roland M, Campbell J. 2015.	Primary care.	Investigate differences in HRQoL (EQ-5D scores) associated with combinations of multimorbidity after adjusting for age, gender, ethnicity, socio-economic deprivation and the presence of a recent illness or injury.	Patients with multi-morbid diabetes, arthritis, neurological, or long-term mental health problems have significantly lower quality of life than other people.	None.
Managing depression in people with multimorbidity: A qualitative evaluation of an integrated collaborative care model. Knowles SE, Chew-Graham C, Adeyemi I, Coupe N, Coventry PA. 2015.	Primary care.	To report the results of a nested qualitative study within the COINCIDE trial, which examined: a) How the collaborative care model was implemented by usual care providers in a UK setting; b) How patients and providers understood and experienced the integration of mental and physical health care.	For complex patients with physical-mental multimorbidity, collaborative care is best understood as a way of organising services to meet their distinct needs. There is a need to develop pragmatic and flexible models of collaborative care that meet the needs of this patient cohort to address the policy objectives of ‘No Health Without Mental Health’.	1
Integrated services for older people – the key to unlock our health and care services and improve the quality of care? Oliver D. 2015.	Multiple contexts.	How integration might help support people in need of health and social care and improve their experience and outcomes.	Many different approaches to integrating services and many definitions, it is not appropriate to dictate “one size fits all” solutions. They must be tailored to local circumstances.	1
The NHS 5 year forward view: lessons from the United States in developing new care models. Shortell SM, Addicott R, Walsh N, Ham C. 2015.	Multiple contexts.	An examination of two of the models proposed by NHS England and discuss how experience from the USA may help inform how they are implemented.	The benefits of integrated care occur primarily through clinical integration rather than organisational integration. The mixed experience of integrated systems and ACOs in the USA points to opportunities and challenges for GPs and specialists working to implement the NHS forward view.	2
Deploying telehealth with sheltered housing tenants living with COPD: a qualitative case study. Bailey C, Cook G, Herman, L, McMillan C, Rose J, Marston R, Binks E, Barron E. 2015.	Local government.	Explore the training and capacity building needed to develop a workforce/older person, telehealth partnership and service that is integrated within existing health, social care and housing services.	Service users and workforces need to work together to provide flexible telehealth monitoring, that in the longer term, may improve service user quality of life.	1
Person centred coordinated care: where does the QOF point us? McShane M, Mitchell E. 2015.	Primary care.	Examine the validity of the Quality and Outcomes Framework and suggest how it should change in the future.	To contribute to the goal of person-centred coordinated care, any financial incentives should be designed to align across primary, secondary, and tertiary care, as well as with the wider health and social care sectors.	None.
Multi-Morbidity in Hospitalised Older Patients: Who Are the Complex Elderly? Ruiz M, Bottle A, Long S, Aylin P. 2015.	Multiple contexts.	Empirically identify the complex elderly patient based on degree of multi-morbidity.	Identification of multi-morbidity patterns can help to predict the needs of the older patient and improve resource provision.	None.
Multimorbidity - older adults need health care that can count past one. Banerjee S. 2015.	Multiple contexts.	Study the healthcare needs of older people with multimorbidity, and the extent to which these needs are met.	Develop a system that works for multimorbidity, and create policy, commissioning, services, research, and education to deliver good quality care to patients with more than one condition.	1
Putting integrated care into practice: the North West London experience. Wistow G, Gaskins M, Holder H, Smith J. 2015.	Primary care. Secondary care.	Summarise progress to date and offer feedback on pilot integrated care systems.	National barriers included: difficulty obtaining data sharing agreements, information governance arrangements; separate payment structures; separate governance structures; balance between collective leadership and local autonomy difficult to strike; change in leadership of programme during development weakened strategic management capacity; led by NHS commissioners, not social care, and tends to reflect NHS interests.	2
Early evaluation of the Integrated Care and Support Pioneers Programme: Final Report. Erens B. 2015.	Primary care. Secondary care. Local services.	Evaluation of the Integrated Care and Support Pioneer programme.	Limited evidence found to support integration.	3
An evidence synthesis of the international knowledge base for new care models to inform and mobilise knowledge for multispecialty community providers (MCPs). Turner A, Mulla A, Booth A, Aldridge S, Stevens S, Battye F, Spilsbury P. 2016.	Multispecialty community providers.	Provide decision makers in health and social care with a practical evidence base relating to the MCP model of care.	MCP seeks to overcome the limitations of current models of care based around single condition-focused pathways, in contrast to patient-focused delivery, which offers greater continuity of care in recognition of complex needs and multimorbidity.	3
Behavioural health consultants in integrated primary care teams: A model for future care. Dale H, Lee A. 2016.	Primary care.	To present the view that integrated care models that incorporate behavioural health care are part of the healthcare solution.	Fully integrated model of behavioural and psychological expertise within a reimagined primary care team, shows promise for addressing the on-going pressures faced in UK general practice and psychological services.	1
The Organisation of Care for People With Multimorbidity in General Practice: An Exploratory Case Study of Service Delivery. Lewis RA. 2016.	Primary care.	Explore service provision for people with multimorbidity in general practice. It considers three research questions: how services are organised; why they are configured in this way; the impact this organisation has on delivery.	To improve outcomes for people with multimorbidity, improving clinical care alone is not as effective as simultaneously improving the organisation or design of services across the whole system of provision.	3
New care models: emerging innovations in governance and organisational form. Collins B. 2016.	Primary care.	Examining different approaches undertaken by MCP and PACS Vanguards to contracting, governance and other organisational infrastructure at five sites across England.	Successful care models are based on trusting relationships and collaborative organisational cultures, often developed over time, which enable clinical teams and organisational leaders to work together effectively. The challenge is how to build clinical collaboration and system leadership in a statutory context that was not designed for this purpose.	2
Harnessing social action to support older people. Georghiou T, Ariti C, Davies M, Arora S, Bhatia T, Bardsley M. Thorlby R. 2016.	Primary care. Social care.	Evaluate seven social action projects funded by Cabinet Office, NHS England, Monitor and Adult Social Services.	Volunteer services helped unmet needs, especially around feelings of isolation; increased productivity and satisfaction of health and social care staff; families and carers benefited; rewarding for volunteers; community scheme associated with higher levels of hospital use; discharge scheme did not reduce time to discharge; A&E scheme did reduce admissions from A&E to hospital.	1
Real lives. Listening to the voices of people who use social care. Hall P, Holder H. 2016.	Social care.	Exploring the experiences of people over-65 s who recently engaged with social care to understand and exemplify the human cost of changes happening within the system.	For the social care system to continue to support people going forward, a renewed national debate about how we pay for and provide care is needed. Without change it seems inevitable that unpaid carers will be expected to do more. More individuals will be required to pay for their care.	1
Untapped Potential: Bringing the voluntary sector’s strengths to health and care transformation. Bull D, Joy I, Weston A, Bagwell S. 2016.	Multiple contexts.	Aims to show that the Voluntary and Community Sector not only support its beneficiaries, but also works to deliver the improvements in health and wellbeing, productivity and efficiency that the health and care system needs to replicate at pace and scale.	The current model of health and care is unsustainable. This presents an opportunity to redesign systems to focus on holistic, integrated, preventative and person-centred care. Partnership between charities and statutory organisations can build a health and social care system which is and fit for purpose.	2
Integrated primary and acute care systems (PACS): Describing the care model and the business model. NHS England. 2016.	Primary care. Secondary care. Community services.	This framework document uses the learning from the nine PACS vanguards to support local health and care systems planning to implement a PACS model.	The evidence suggests five crucial elements for success of the PACS model: A commitment to partnership working; A data-driven care model that organises care around population segments; Integrated neighbourhood health and care teams; Flexible use of workforce and technology; Contracting, funding and organisational model designed to deliver the population-based care model.	3
Developing a user reported measure of care co-ordination. Crump H, King J, Graham C, Thorlby R, Raleigh V, Redding D, Goodwin N. 2017.	Multiple contexts.	The study designed and tested a survey tool to capture the experiences of older people with chronic conditions regarding how well their health and social care was co-ordinated.	The growing focus on care co-ordination demonstrates the need for a tool that early results suggest may have a contributed to capturing experiences of patients accessing care across organisational and professional boundaries, to inform improvement of care co-ordination from a patient perspective.	1
Multimorbidity and Integrated Care. Stokes J. 2017.	Multiple contexts.	Exploring the effectiveness of current models of integrated care. The extent to which there are differential effects of integrated care for different types of multimorbidity.	Integrated care, in its current manifestation, is not a silver bullet that will enable health systems to simultaneously accomplish better health outcomes for those with LTC and multimorbidity, while increasing their satisfaction with services and reducing costs.	3
Creating and facilitating change for Person-Centred Coordinated Care (P3C): The development of the Organisational Change Tool (P3C - OCT). Horrell J, Lloyd H, Sugavanam T, Close J, Byng R. 2017.	Multiple contexts.	Examination of current policy, key literature and NHS guidelines, together with stakeholder involvement to identify domains, subdomains and component activities required to deliver patient-centred co-ordinated care.	Ongoing interrogation of the interaction between domains/subdomains (question items) and components (response codes) from implementation data will allow development of a more comprehensive theory of what works for whom and in what situations, to best accomplish patient-centre co-ordinated care.	2
Shaping innovations in long-term care for stroke survivors with multimorbidity through stakeholder engagement. Sadler E, Porat T, Marshall I, Hoang U, Curcin V, Wolfe CDA, McKevitt C. 2017.	Multiple contexts.	Develop a process of engaging stakeholders in the use of clinical and research data to co-produce potential solutions to improve long-term care for stroke survivors with multimorbidity.	Stakeholder engagement to identify data-driven solutions is feasible but requires resources.	None.
Better value primary care is needed now more than ever. Watson J, Salisbury C, Jani A, Gray M, McKinstry B, Rosen R. 2017.	Primary care.	An exploration of how the value-based healthcare framework inform decisions about allocating resources, and the importance of good evidence not only for patient treatment but for the organisation of health services.	Effective primary care is essential to deliver high value care, but change needs to be driven by evidence based policy and investment.	3
Enhancing Health and Wellbeing in Dementia: A Person-Centred Integrated Care Approach. Rahman S. 2017.	No specific context.	To challenge the idea that little can be done to improve dementia care and set out practical thinking around moving towards integrated person-centred working.	Key underpinnings of integrated care for wellbeing when living with dementia, including technology, staff performance, leadership, and intelligent regulation of services.	1
Integrated Working for Enhanced Health Care in English Nursing Homes. Cook G, McNall A, Thompson J, Hodgson P, Shaw L, Cowie D. 2017.	Nursing home sector.	Explore views and experiences of practitioners, social care officers, and carers involved in the enhanced health care in care home programme, to develop understanding of the service delivery model and associated workforce needs for the provision of health care to older residents.	The programme provides a whole system approach to the delivery of proactive and responsive care for nursing home residents. The service model enables information exchange across organisational and professional boundaries.	2
Delivering person-centred holistic care for older people. Beech R. Ong BN, Jones S, Edwards V. 2017.	Multiple contexts.	An evaluated case study of the Wellbeing Coordinator (WBC) service in Cheshire. WBCs are non-clinical members of the GP surgery or hospital team who offer advice and support to help people with LTC and unmet social needs remain independent at home.	WBC complements medical approaches to supporting people with complex health and social care problems. Users reported improvements in their wellbeing, access to social networks, and maintenance of social identity and valued activities. Health and social care professionals recognised the value of the service.	None.
Enhanced health in care homes: Learning from experiences so far. Baylis A, Perks-Baker S. 2017.	Principally Care Homes, includes primary care interface.	Help care homes and NHS providers join up and co-ordinate services locally, and manager the complexities involved.	Enhanced health in care homes is achievable in England without extra funding.	3
Primary Care Home Evaluating a new model of primary care. Kumpunen S, Rosen R, Kossarova L, Sherlaw-Johnson C. 2017.	Primary care. Secondary care.	Understand how ‘primary care home’ integrated models were built; advise on evaluation approaches; share learning across sites.	All models targeted local health needs and weaknesses in local services.	1
Hope over experience: still trying to bridge the divide in health and social care. Wistow G. 2017.	Healthcare. Social care. Local government.	Discuss the divide in health and social care, specifically local government and how this might be bridged.	Little progress has been made on integration. Local government is critical, seeing it as all about NHS bodies and financial control, with local authorities An optional add on, when a whole system solution remains the answer.	1
Improving Hospital at Home for frail older people: insights from a quality improvement project to achieve change across regional health and social care sectors. Pearson M, Hemsley A, Blackwell R, Pegg L, Custerson L. 2017.	Secondary care. Social and community care.	To change practice in order to deliver a Hospital at Home programme (admission avoidance and early supported discharge) for frail older people across a regional commissioning area.	Against a backdrop of intense financial pressures, significant community bed closures, and difficult relations between hospital and community services, outcomes remained stable.	1
Long-term clinical and cost-effectiveness of collaborative care (versus usual care) for people with mental-physical multimorbidity: cluster-randomised trial. Camacho EM, Davies LM, Hann M, Small N, Bower P, Chew-Graham C, Baguely C, Gask L, Dickens CM, Lovell K, Waheed W, Gibbons CJ, Coventry P. 2018.	Primary care.	Explore the long-term (24-month) effectiveness and cost-effectiveness of collaborative care in people with mental-physical multimorbidity.	In the long term, collaborative care reduces depression and is potentially cost-effective at internationally accepted willingness to-pay thresholds.	None.
Management of multimorbidity using a patient-centred care model: a pragmatic cluster-randomised trial of the 3D approach. Salisbury C, Man MS, Bower P, Guthrie B, Chaplin K, Gaunt DM, Brookes S, Fitzpatrick B, Gardner C, Hollinghurst S, Lee V, McLeod J, Mann C, Moffat KR, Mercer SW. 2018.	Primary care.	Examine if patient-centred, so-called 3D intervention (based on dimensions of health, depression, and drugs) for patients with multimorbidity would improve their health-related quality of life.	The 3D intervention did not improve patients’ quality of life.	1
Elements of integrated care approaches for older people: A review of reviews. Briggs AM, Valentijn PP, Thiyagarajan JA, Araujo de Carvalho I. 2018.	No specific context.	To identify and describe the key elements of integrated care models for elderly people reported in the literature.	Evidence of elements of integrated care for older people focuses particularly on micro clinical care integration processes, while there is a relative lack of information regarding the meso organisational and macro system-level care integration strategies.	3
Understanding care navigation by older adults with multimorbidity: Mixed-methods study using social network and framework analyses. Vos J, Gerling K, Linehan C, Siriwardena AN, Windle K. 2018.	Primary care. Secondary care. Social care.	Design technology to assist people with LTCs in navigating health and social care systems.	Quality of care is dependent on the determination and ability of patients. Those with less determination and fewer organisational skills experience worse care. Technology must aim to fulfil these coordination functions to ensure care is equitable for those who need it.	2
Supporting shared decision making for older people with multiple health and social care needs: A realist synthesis. Bunn F, Goodman C, Russell B, Wilson P, Manthorpe J, Rait G, Hodkinson I, Durand MA. 2018.	Multiple contexts.	Provide a context-relevant understanding of how models of shared decision-making might work for older people with multiple health and care needs, and how they might be applied to integrated care models.	Models of shared decision-making for older people with complex health and care needs should be conceptualised as a series of conversations that patients, and their family carers, may have with a variety of different health and care professionals.	2
‘Trying to put a square peg into a round hole’: a qualitative study of healthcare professionals’ views of integrating complementary medicine into primary care for musculoskeletal and mental health comorbidity. Sharp D, Lorenc A, Feder G, Little P, Hollinghurst S, Mercer S, MacPherson H. 2018.	Primary care.	Explore professionals’ experiences and views of CAM (Integration of conventional with complementary approaches) for comorbid patients and the potential for integration into UK primary care.	CAM has the potential to help the NHS in treating the burden of MSK and mental health comorbidity. Selective incorporation using traditional referral from primary care to CAM may be the most feasible model.	1
Primary care redesign for person-centred care: delivering an international generalist revolution. Reeve J. 2018.	Primary care.	To draw on the UK Society for Academic Primary Care’s model of blue sky thinking to propose a ‘Dangerous Idea’: an idea that challenges the status quo but with a commitment to action.	To achieve person-centred healthcare, we need to redesign healthcare around the expertise of the generalist clinician in making whole-person, goal oriented clinical decisions.	1
Is telephone health coaching a useful population health strategy for supporting older people with multimorbidity? An evaluation of reach, effectiveness and cost-effectiveness using a ‘trial within a cohort’. Panagioti M, Reeves D, Meacock R, Parkinson B, Lovell K, Hann M, Howells K, Blakemore A, Riste L, Coventry P, Blakeman T, Sidaway M, Bower P. 2018.	Primary care.	Examine health coaching, which is ‘a regular series of phone calls between patient and health professional to provide support and encouragement to promote healthy behaviours’.	Health coaching in multimorbid patients did not lead to significant benefits on the primary measures of patient-reported outcome. The optimal role of this model of care within integrated care systems for patients with multiple LTC remains unclear.	1
Enacting person-centredness in integrated care: A qualitative study of practice and perspectives within multidisciplinary groups in the care of older people. Riste LK, Coventry PA, Reilly ST, Bower P, Sanders C. 2018.	Multiple contexts.	Describe how person-centred care is enacted within multidisciplinary groups (MDGs) created as part of a new service integrating health and social care for older people.	Three themes were identified that impacted on person-centred care: the structural context of MDGs enabling person-centred care; interaction of staff and knowledge sharing during MDG meetings; direct staff involvement of the person outside the MDG discussion.	1
A year of integrated care systems: reviewing the journey so far. Charles A, Wenzel L, Kershaw M, Ham C, Walsh N. 2018.	Primary care. Secondary care. Local Government. Community providers. Mental health.	Assess features, changes in services, leadership, organisation, regulation of developing integrated care systems on local and national level; understand factors helping and hindering progress in developing integrated care systems.	Enablers of integrated care: collaborative relationships, shared vision/purpose, system and clinical leadership. Barriers to integrated care: legislative context not supportive of system working; legacy of competitive behaviours between organisations; frequently changing language; funding pressures hindering, as well as helping, progress.	3
Managing the hospital and social care interface: Interventions targeting older adults. Holder H, Kumpunen S, Castle-Clarke S, Lombardo S. 2018.	Secondary care. Social care.	Explore actions and strategies providers and commissioners employed to improve interface between secondary and social care, especially hospitals.	Focusing on delayed transfer of care does not address wider issues facing health and social care, and focusing on this issue causes confrontation and negatively impacts local relationships; small- and large-scale organisational change are both required.	2
Advancing Integrated Care in England: A Practical Path for Care Transformation. McClellan M, Udayakumar K, Thoumi A, Gonzalez-Smith J. 2018.	Multiple contexts.	Assess effective and practical paths to accelerate the adoption of better integrated, higher-value care.	Barriers to integrated care included: tight budgets, pressure to meet performance targets in the short term, conflicting requirements among regulatory entities with overlapping oversight, and the perception of frequently shifting policy objectives.	3
Priorities for the Plan. The long-term NHS plan and beyond: Views from leaders in charities and voice organisations. Redding D. 2018.	Primary care. Secondary care.	Discusses the need for new models of integrated care, especially the importance of person-centred care.	So long as separate performance requirements and perverse funding incentives remain in place, system-centred reform will always trump a person-centred approach.	1
Multimorbidity - Understanding the challenge. Aiden H. 2018.	Third Sector. Think Tanks. Primary care. Universities.	Understand and respond to one of the largest and most complex challenges facing modern health and care systems.	A better understanding of the experiences, wishes and goals of people with multiple conditions will help inform the design and delivery of health and social care services.	3
Beyond barriers. How older people move between health and social care in England. Care Quality Commission. 2018.	Primary care. Community health services. Social care. Third sector.	Review local health and social care systems in 20 local authority areas.	People experience the best care when they and organisations work together to overcome the fragmentation of the health and social care system and coordinate personalised care around individuals.	2
A patient-centred intervention to improve the management of multimorbidity in general practice: the 3D RCT. Salisbury C, Man MS, Chaplin K, Mann C, Bower P, Brookes S, Duncan P, Fitzpatrick B, Gardner C, Gaunt DM, Guthrie B, Hollinghurst S, Kadir B, Lee V, McLeod J, Mercer SW, Moffat KR, Moody E, Rafi I, Robinson R, Shaw A, Thorn J. 2019.	Primary care.	Assess a care model that incorporated all strategies recommended by current guidelines.	Both patients and staff welcomed having more time, continuity of care and the patient-centred approach. The economic analysis found no meaningful differences between the intervention and usual care in either quality-adjusted life-years or costs.	1
Interventions for involving older patients with multi-morbidity in decision-making during primary care consultations. Butterworth JE, Hays R, McDonagh ST, Richards SH, Bower P, Campbell J. 2019.	Primary care.	Review available evidence about the effects of interventions intended to involve older people with more than one long-term health problem in decision-making about their health care during primary care consultations.	No consistent evidence that interventions for involving older people with more than one long-term health problem in decision-making about their health care can improve their self-rated health or healthcare engagement. The interventions make little or no difference in patients’ quality of life.	1
Service user, carer and provider perspectives on integrated care for older people with frailty, and factors perceived to facilitate and hinder implementation: A narrative synthesis. Sadler E, Potterton V, Anderson R, Khadjesari Z, Sheehan K, Butt F, Sevdalis N, Sandall J. 2019.	Multiple contexts.	Explore service user, carer and provider views on integrated care for older people with frailty, and factors perceived to facilitate and hinder implementation, to draw out implications for policy and practice.	Findings highlight the importance of addressing organisational and system level components of integrated care and factors influencing implementation. Greater attention needs to be placed on collaboratively involving service users, carers and providers to improve the co-design and implementation.	3
Does pooling health and social care budgets reduce hospital use and lower costs? Stokes, J. Lau YS, Kristensen SR, Sutton M. 2019.	Secondary care.	This study examined variation in timing of uptake to examine the short- (1 year) and intermediate-term (up to 2 years) effects of the Better Care Fund on 7 measures of hospital use and costs from a cohort of 14.4 million patients.	In the short term, pooling health and social care budgets alone does not appear to reduce hospital use nor costs but does appear to additionally stimulate integration activity.	2
Does a social prescribing ‘holistic’ link-worker for older people with complex, multimorbidity improve well-being and frailty and reduce health and social care use and costs? A 12-month before-and-after evaluation. Elston J, Gradinger F, Asthana S, Lilley-Woolnough C, Wroe S, Harman H, Byng R. 2019.	Primary care. Secondary care.	Evaluate the impact of ‘holistic’ link-workers on service users’ well-being, activation and frailty, and their use of health and social care services and the associated costs.	Holistic link-workers improved quality of life, patient activation and reduced frailty in a complex cohort with multiple LTC. Just under half of referrals saw a decrease or no change in cost and activity after 12 months.	1
An Investigation into the Awareness, Demand and Use of Community Pharmacy Services for People with Long-term Conditions. Hindi AMK. 2019.	Primary care. Community services.	Explore and identify ways to improve low awareness, demand and use of community pharmacy services which may benefit patients with LTCs.	Community pharmacies have potential to offer more support for patients with LTCs but further developments are needed to fully integrate community pharmacy services within patient primary care pathways.	1
Leading for integrated care: ‘If you think competition is hard, you should try collaboration’. Timmins N. 2019.	Multiple contexts.	Reflect the views of 16 chairs and leads of both STPs and ICSs on the challenges involved.	Better integrated care requires the dilution or destruction of long-standing barriers between hospitals, GP practices, community services and social care. This requires system leadership.	2
Payments and contracting for integrated care: The false promise of the self-improving health system. Collins B. 2019.	Primary care. Secondary care.	Explore if new funding models incentivise effective delivery of integrated care.	Current funding for integrated health care systems relies on incentives for performance. There are no standardised ways to measure success in delivering integrated care, these metrics may not drive desirable performance.	2
Age UK’s Care Programme Personalised Integrated Evaluation of Impact on Hospital Activity. Georghiou T, Eilsi K. 2019.	Healthcare. Social care. Voluntary services.	Assessment of whether the PICP intervention improve outcomes.	Personal integrated care programme (AGE UK) is associated with increased admission rates and increased healthcare costs.	1
Effects of participating in community assets on quality of life and costs of care: study of older people in England. Munford LA, Wilding A, Bower P, Sutton M. 2020.	Community.	Generate evidence on whether expansion of social prescribing to charity, voluntary and community groups is associated with better quality of life or lower care costs.	The results support the inclusion of community assets as part of an integrated care model for older patients.	None.
Leadership for integrated care: a case study. Kozlowska O, Gombau GS, Rea R. 2020.	Primary care. Specialist care.	Explore the complexities of leadership in an integrated care project and aims to understand what leadership arrangements are needed to enable service transformation.	Integration was supported in the narratives of the system and organisational leaders but there were multiple challenges, e.g. insufficient support by the system level leadership for the local leadership, insufficient organisational support for (clinical) leadership within the transformation team.	1
Integrated primary care for patients with mental and physical multimorbidity: cluster randomised controlled trial of collaborative care for patients with depression comorbid with diabetes or cardiovascular disease. Coventry P, Lovell K, Dickens C, Bower P, Chew-Graham C, McElvenny D, Hann M, Cherrington A, Garrett C, Gibbons CJ, Baguley C, Roughley K, Adeyemi I, Reeves D, Waheed W, Gask L. 2015.	Primary care.	Test the effectiveness of an integrated collaborative care model for people with depression and long-term physical conditions.	Collaborative care that incorporates brief low intensity psychological therapy delivered in partnership with practice nurses in primary care can reduce depression and improve self-management of chronic disease in people with mental and physical multimorbidity.	2
The Real-World Problem of Care Coordination: A Longitudinal Qualitative Study with Patients Living with Advanced Progressive Illness and Their Unpaid Caregivers. Daveson BA, Harding R, Shipman C, Mason BL, Epiphaniou E, Higginson IJ, Ellis-Smith C, Henson L, Munday D, Nanton V, Dale JR, Boyd K, Worth A, Barclay S, Donaldson A, Murray S. 2014.	Primary care. Secondary care.	Develop a model of care coordination for patients living with advanced progressive illness and their unpaid caregivers, and to understand their perspective regarding care coordination.	Within the midst of advanced progressive illness, coordination is a shared and complex intervention involving relational, structural and information components. These findings can be used to help avoid oversimplifying a real-world problem, such as care coordination.	2
Integrated care. What is it? Does it work? What does it mean for the NHS? Ham C., Curry, N. 2011.	Healthcare.	Describe the different forms of integrated care and to summarise evidence on their impact.	Organisational integration appears to be neither necessary nor sufficient to deliver the benefits of integrated care, notwithstanding the achievements of integrated systems such as the Veterans Health Administration.	3
Integrated care in Northern Ireland, Scotland and Wales: Lessons for England. Ham C, Heenan D, Longley M, Steel DR. 2013.	Healthcare. Social care.	To describe the approach taken to integrated care in Northern Ireland, Scotland and Wales to draw out the lessons for England.	Integrating health and social care within the same structures may have the unintended consequence of social care becoming subservient to health care.	3

### Level of integration

The number of studies that considered integration were counted at each level as set out in the conceptual framework (i.e. macro, meso or micro-level). There were 7% of studies that considered integration at the macro-level, 5% at meso-level and 30% at micro-level. Thirty-five per cent of the articles considered integration at all three levels. However, the combined number of studies focused on either one or both of micro and meso levels was 52%.

### Summary of themes amongst included studies

Three themes were identified from the analysis, which summarised current research and progress on integrated primary care and social services for older adults with multimorbidity in England: (1) a diverse focus on multi-level vs. multi-sector integration; (2) time needed for integration to embed; and (3) seeking structural integration while applying local flexibility. Each of these is described in turn below.

#### Multi-level and multi-sector integration

Several articles described previous research and clinical provision in primary care or social services for older adults with multimorbidity in England [17–20]. These were often concerned with particular sectors (e.g. primary care) or scales of integration (e.g. clinical level), rather than whole-systems reform [21–27]. Studies focused on improving specific dimensions of integration such as leadership [17, 28], care models [22, 29–32] or considered integrated working from the perspective of one or two levels of integration [33–36], most frequently the micro-scale or the micro/meso-scales together. Little evidence was found of functional or normative forms of, or approaches to, integration as described in the Rainbow Model of Integrated Care conceptual framework [12]. Studies from 1996 onwards repeatedly stressed the need for more multi-level, systemic and comprehensive integration [37, 38], although limited evidence was found of significant progress in achieving this ambition over the last two decades. A prevalent theme was the urgent requirement to mitigate or remove long-standing barriers to integration, such as incompatible record sharing systems and inadequate information sharing processes between sectors [39–43]; ‘siloed’ thinking in service provider organisations [44–46]; poor communication among health and social care professionals, both internally within their organisations and across sectoral boundaries [47–49]. There was an increasing emphasis on the need to tackle wider determinants of population health with suggestions that to achieve this, it is necessary to go beyond primary care and social services to include hospitals, GP community services, voluntary sectors and local government partners [10, 32, 48, 50, 51]. There was a growing recognition in more recent literature that improving clinical care in one or two sectors may not be as effective as simultaneously improving organisation or design across services, as one system of provision [43]. Solutions that were proposed emphasised the need for system-wide leadership across all scales, alongside a shared vision of integrated working across sectors [52–55]. There was evidence highlighting the importance of the quality and style of organisational leadership, both in terms of delivering change and maintaining an integrated approach to service delivery [9, 28, 56]. Few examples were found of where this approach had led to individual and local successes, and widespread evaluation and evidence of application was very limited [31, 34, 47, 57].

#### Time for integration to embed

A number of studies highlighted that integration requires time to allow new structures and relationships to develop and bed-in. Integrated care programmes take years to establish and need sufficient time to allow new care models to fully mature [19, 58–60]. Effective and enduring integration is ‘the result of a long-term process, facilitated by key local leaders, during which the capability and legitimacy of new ways of working is built up over time’ [50]. The King’s Fund report of the Vanguards concluded that the most successful models of integrated care are built on ‘trusting relationships and collaborative organisational cultures’, which ‘often developed over time,’ enabling ‘clinical teams as well as key organisational leaders to work together effectively’ [29]. This highlights the importance of time in supporting and sustaining long term individual, inter-professional and co-operative organisational relationships and cultures, which are a key component of normative integration [12], understood as “the development and maintenance of a common frame of reference (i.e., shared mission, vision, values and culture) between organizations, professional groups and individuals” [61].

Some studies suggest that the answer to the challenges of integration may lie in persistence and perseverance over several years to enable integrated care programmes to achieve their ‘objectives and become self-sustaining’ [53, 62, 63]. This appeared to be influenced by the sustained commitment of key partners and the ‘longevity of the senior leadership’ [57]. The challenge in the next phase of integrated care reform is ‘building clinical collaboration and system leadership in a statutory context’ that is ‘not designed for this purpose’, [29] alongside policymakers providing the necessary time for integrated care programmes to ‘evolve and mature’, [64] rather than moving onto the next new policy initiative.

## Structure with flexibility

The scoping review identified inherent tensions between top-down and bottom-up driven approaches to integrated care, in particular, having in place a single comprehensive ‘whole-systems’ structure combined with local flexibilities. Studies suggested that integration should be implemented within a clear framework and a set of higher-level principles that allows for both macro-level systems-wide strategic management and oversight, combined with local autonomy and flexibility, described as ‘structured flexibility’ [19, 65–68]. The benefit of holistic systems-wide approaches is that they ‘tend to be more strategic with clearer paths for scaling up, compared to ‘bottom-up’ approaches driven by highly motivated individuals at the micro-level’ [56]. Nevertheless, a whole-systems strategy requires a twin-track approach [55], with ‘leadership from the bottom up’ driven by staff who are ‘empowered to integrate services where they see the need’ [53]. Mechanisms for horizontal integration (structures, strategies and practices that connect care across the same level in the system) [12], were also seen as essential ‘at each organisational level (for example whole systems, community and individual levels). Vertical mechanisms (structures, strategies and practices which link together services up and down the different scales of the system) are also necessary ‘to integrate the various levels’ [37, 42]. Successful examples of integrated care in the NHS indicate that when this is ‘pursued at all levels’, it could ‘overcome the risks of fragmentation, and of ‘service users falling between the cracks’ of care [69]. Critically however, the studies included in the review suggested that any programme of integrated care must be based on an understanding that ‘as barriers to integration are systemic in organisations designed for separation rather than integration and the historic paradigms of building bridges and tearing down walls is inherently flawed, and of limited effectiveness: a better metaphor is one of weaving integration into the fabric of organisational life’ [37].

## Discussion

This scoping review aimed to summarise current evidence, clinical provision and progress on integrated primary care and social services for older adults experiencing multimorbidity in England. The findings highlight a paucity of research evidence and clinical practice pursuing multi-level or multi-sector integration across services. Furthermore, existing literature in this field are often limited to individual sectors [21–27]. The value of considering primary care and social services alongside local government, third sector and secondary care organisations in tackling the broader determinants of population health was frequently emphasised [10, 32, 48, 50, 51]. In addition, several studies highlighted that integration requires time [19, 58–60] to allow new structures and relationships to evolve and mature. The recent development of the Primary Care Network (PCN) Maturity Matrix, as a methodology and systems development approach, has potential to address this issue. PCN sets out a developmental pathway or framework to guide systems leaders, which focuses attention on the importance of allowing integrated care programmes sufficient time to bed-in and reach a state of maturity [70, 71]. The scoping review identified inherent tensions between top-down and bottom-up driven dimensions of integrated care reform. The evidence gathered by this scoping review suggests that addressing this dichotomy requires both whole system structures, which allow for local flexibilities [19, 65–68].

This study was scoping in nature, thus allowing a rapid capture of a broad range of information on integration between primary care and social services in older adults with multimorbidity. It did not aim to answer a strictly defined research question and as a result, broad inclusion criteria were adopted, which allowed for the inclusion of a wide-range of study designs and grey literature to permit a higher-level overview of this research area and the related clinical provision. As much of social care delivery takes place outside of the NHS, and as a consequence of the relative paucity of research in this field, this approach was necessary to capture the diverse range of existing work in the field. There were high levels of heterogeneity amongst the study designs and settings which is a strength of this work but also challenging to collate and summarise comprehensively. The quality of the evidence presented was not assessed and those articles not written in English were excluded. As the study was focused on England, it is unlikely that non-English language articles would have substantially altered the results.

This scoping review is one of the first to examine the literature on integration between primary care and social services, with a particular focus on England. The findings are consistent with previous evidence outside of a primary care context, which highlights the need for greater consideration of wider health determinants in managing the increasingly diverse needs of older adults with multimorbidity [72, 73]. Earlier reviews on integrated care have also emphasised the need for more multidisciplinary and multi-sector co-operation within a single over-arching system [74]. The study highlighted that this system must incorporate traditional health and social care services, alongside voluntary, private and government organisations. Furthermore, the scoping review emphasised the value of time in allowing integrated care to embed. Although some previous reviews and policy calls argue that a more rapid and urgent pace is needed for integration due to rising demand, this scoping review suggests that a slower process of change is perhaps necessary to permit successful and long-lasting implementation at the local level [7]. This has been highlighted by a previous scoping review although it was not specific to primary care or social services [50, 59, 63, 74].

Finally, the tensions identified between top-down and bottom-up integrated care reform [56, 65] and related calls for whole system structures of integration allowing for local flexibilities, has been articulated in government policy but not yet operationalised [55]. To support this, the next steps will need to go beyond a scoping review towards more robust service evaluation and trials of whole-system multi-sector and multi-level integration interventions that address both clinical and social need.

## Conclusions

This scoping review aimed to summarise current evidence, clinical provision and progress towards integrated primary care and social services for older adults with multimorbidity in England. It found studies describing individual sectors, which mainly focused on process improvements, while there was limited evidence of improved outcomes or resource use, nor evidence of provision or progress towards multi-level and multi-sector integration across services for older adults with multimorbidity. Wider determinants of population health are important, suggesting that integration that goes beyond primary care and social services to encompass a truly whole system approach across sectors is likely to be necessary to effectively address the needs of older adults with multimorbidity. This may take time to establish and will require local input. Further research evidence is required to support operationalising this approach and to examine the feasibility of implementing such a system within existing structures.

## Supplementary Information

**Additional file 1.**

## Data Availability

Data used during the current study are available from the corresponding author on reasonable request.
