# Supplementary material for: Integrated primary care and social services for older adults with multimorbidity in England: a scoping review
Source: BMC Geriatr. 2021 Dec 3;21:674. doi: 10.1186/s12877-021-02618-8 (PMC8642958; doi:10.1186/s12877-021-02618-8)
Supplement: Supplementary file 1 — Additional file 1. [file 12877_2021_2618_MOESM1_ESM.docx]

**Supplementary material:**

**Example of searches**

| **MeSH and search terms** | **Databases searched** | **Filters** | **Number of sources identified** |
| --- | --- | --- | --- |
| ((((("integrated"[All Fields]) AND ("social care"[All Fields])) AND ("multimorbidity"[All Fields]))  (("integrated"[All Fields]) AND ("multimorbidity"[All Fields]) | Medline (Pubmed) | All dates searched.  Language: restricted to English | 366 |
| (integrated care and multimorbidity).mp. [mp=title, abstract, heading word, drug trade name, original title, device manufacturer, drug manufacturer, device trade name, keyword, floating subheading word, candidate term word] | Embase | All dates searched.  Language: restricted to English | 96 |
| ‘’integrated’’ in Title Abstract Keyword AND ‘’social care’’ in Title Abstract Keyword AND ‘’multimorbidity’’ Title, Abstract Keyword (Word variations have been searched)  MeSH descriptor: [Delivery of Health Care, Integrated] AND [Multimorbidity] | Cochrane Library | All dates searched.  Language: restricted to English | 394 |
| [Integrated care AND Social Care AND Multimorbidity] using Topic Search  [Integrated care AND Multimorbidity] using Topic Search | Web of Science | All dates searched.  Language: restricted to English | 128  417 |
| Integrated care AND social care AND multimorbidity  Integrated care AND multimorbidity | Cumulative Index to Nursing and Allied Health Literature  (CINAHL) | All dates searched.  Language: restricted to English | 2036  2,577 |
| [Integrated care AND Social Care AND Multimorbidity] using Topic Search  [Integrated care AND Multimorbidity] using Topic Search | Social Science Citation Indices & Science Citation Indices | All dates searched.  Language: restricted to English | 84  328 |
|  |  |  |  |
| Integrated care  Care of the elderly  Social care and integrated care | Open Grey | All dates searched  Language: restricted to English | 214  810  94 |
| Terms used: Integrated care AND Social Care AND Multimorbidity | Generic grey literature website searches:-  Clinical Commissioning Groups, GP federations, the Department of Health and Social Care, third sector bodies, private organisations who deliver social care and think tanks  Snowball searches from references | Language: restricted to English | 65  47 |

# **Adapted PRISMA Flow Chart Explaining the Study’s Documentary Inclusion Process**

# (Page MJ et al., 2021) [75]

## Screening

## Eligibility

**Records after duplicates removed** (n = 3,923)

**Records titles/abstracts screened** (n = 3,923)

**Records excluded**
(n = 3,114)

**Full-text articles assessed for eligibility** (n = 809)

**Records identified through database searching** (n = 7,656)

**Articles included in the review** (n = 84)

**Full-text articles excluded, with reasons** (n = 700)

Wrong population (n = 268)

International focus (n = 183)

Not related to England (n = 161)

Background article (n = 32)

Maybe some England data (n = 23)

Foreign language (n = 8)

Duplicates (n = 8)

Wrong publication type (n = 6)

No abstract included (n = 6)

Wrong outcome (n = 5)

|  |
| --- |
|  |
|  |
|  |

**In-depth assessment** (n = 109)

Primary international in scope, not England focused (n = 25)

## Identification

## Included
